# Cytokine release syndrome-compatible fever after initiation of local radiotherapy during late-phase epcoritamab therapy in relapsed/refractory diffuse large B-cell lymphoma: a case report

**DOI:** 10.1186/s40780-026-00568-0

**Published:** 2026-03-27

**Authors:** Naoaki Nishimura, Hajime Nakashima, Yuto Ogasa, Kenji Yoshikuni, Kentaro Kohno, Ryosuke Ogawa

**Affiliations:** 1 Department of Pharmacy, Japan Community Health Care Organization (JCHO) Kyushu Hospital, 1-8-1 Kishinoura, Yahatanishi-ku, Kitakyushu, Fukuoka 806-8501 Japan; 2 Department of Hematology and Oncology, Japan Community Health Care Organization (JCHO) Kyushu Hospital, 1-8-1 Kishinoura, Yahatanishi-ku, Kitakyushu, Fukuoka 806-8501 Japan

**Keywords:** Bispecific antibody, Epcoritamab, Radiotherapy, Cytokine release syndrome, Tocilizumab, Diffuse large B-cell lymphoma

## Abstract

**Background:**

Epcoritamab, a subcutaneous CD3×CD20 bispecific antibody, has demonstrated promising activity in relapsed or refractory diffuse large B‑cell lymphoma. Cytokine release syndrome (CRS) is a key adverse event associated with T‑cell–engaging therapies; however, it typically occurs early after treatment initiation and is generally manageable with step‑up dosing and premedication. Evidence regarding the safety of delivering radiotherapy during late-phase epcoritamab therapy remains limited.

**Case presentation:**

A 58-year-old man with diffuse large B-cell lymphoma received epcoritamab. On cycle 8 day 8, local radiotherapy (40 Gy in 20 fractions) was initiated for a residual abdominal lymph node lesion. Approximately 12 h after the second radiotherapy fraction, he developed fever (38 °C) and fatigue without hypotension or hypoxia and without neutropenia, consistent with a CRS-compatible febrile episode (grade 1 by the American Society for Transplantation and Cellular Therapy criteria). Blood cultures were obtained and empiric cefepime with acetaminophen was initiated, resulting in transient defervescence; however, fever recurred approximately 12 h after the third radiotherapy fraction. Blood cultures, (1,3)-β-D-glucan, and galactomannan assays were negative. Given the reproducible temporal association with radiotherapy, a CRS-compatible event was considered, and tocilizumab (8 mg/kg) was administered in line with standard CRS management guidance, with defervescence within 1 h. He remained afebrile thereafter and completed radiotherapy without recurrence.

**Conclusions:**

CRS-compatible fever is uncommon after multiple cycles of epcoritamab. This case suggests a temporal association between radiotherapy and a CRS-compatible febrile episode during late-phase therapy and supports the possibility that radiotherapy acted as an inflammatory trigger. When radiotherapy is delivered during bispecific antibody therapy, fever should prompt concurrent evaluation for infection while keeping CRS in the differential diagnosis, and standard CRS management should be applied promptly when clinically indicated.

## Background

Diffuse large B-cell lymphoma (DLBCL) is an aggressive lymphoma, and outcomes remain poor in relapsed or refractory (R/R) disease. Patients with limited responsiveness to salvage cytotoxic chemotherapy require additional therapeutic options. The treatment landscape for DLBCL has recently been reshaped by bispecific antibodies.

Epcoritamab is a subcutaneous CD3×CD20 bispecific antibody (BsAb) that mediates T-cell–dependent cytotoxicity against malignant B cells and provides an off-the-shelf option for patients who are ineligible for, or refractory to, chimeric antigen receptor (CAR) T-cell therapy [1, 2]. Cytokine release syndrome (CRS) is a characteristic acute toxicity associated with on-target immune activation during T-cell–engaging immunotherapies. CRS reflects systemic inflammation driven by immune-cell activation and cytokine release, including interleukin-6 (IL-6), and is clinically defined by fever with or without hypotension, hypoxemia, and organ dysfunction [3–5]. CRS severity should be graded according to the 2019 American Society for Transplantation and Cellular Therapy (ASTCT) consensus criteria [5]. To mitigate CRS risk, epcoritamab is administered using step-up dosing with standard premedication. In the pivotal large B-cell lymphoma (LBCL) cohort, CRS occurred predominantly after the first full dose on cycle 1 day 15 and was mostly low grade; grade 3 CRS occurred in a small proportion of patients, and CRS typically resolved within a short timeframe with standard management, including tocilizumab when indicated [3, 6]. In extended follow-up of EPCORE NHL-1, most CRS events were confined to early cycles, with the latest reported CRS onset at cycle 4 day 1 and no new CRS events observed thereafter [2]. Accordingly, febrile episodes with CRS-like features occurring in later cycles warrant careful assessment for concurrent triggers (e.g., infection) while maintaining CRS in the differential diagnosis [2, 5].

Here, we report a case in which fever temporally associated with radiotherapy (RT) recurred during late-phase epcoritamab therapy, prompting parallel evaluation for infection while maintaining a CRS-compatible event in the differential diagnosis.

## Case presentation

A 58-year-old man was diagnosed with DLBCL, not otherwise specified (DLBCL, NOS), stage IV in August of year X − 2. He received rituximab plus cyclophosphamide, doxorubicin, vincristine, and prednisone (R-CHOP) for six cycles; however, left axillary lymphadenopathy developed in January of year X − 1, and biopsy confirmed refractory DLBCL. He subsequently received two cycles of rituximab plus etoposide, methylprednisolone, cytarabine, and cisplatin (R-ESHAP) and polatuzumab vedotin plus rituximab, followed by CAR T-cell therapy with lisocabtagene maraleucel in June of year X − 1. No CRS occurred after CAR T-cell therapy. Positron emission tomography–computed tomography in October of year X − 1 showed complete remission; however, relapse was documented on computed tomography in March of year X, and epcoritamab therapy was initiated in March of year X.

**Fig. 1:**
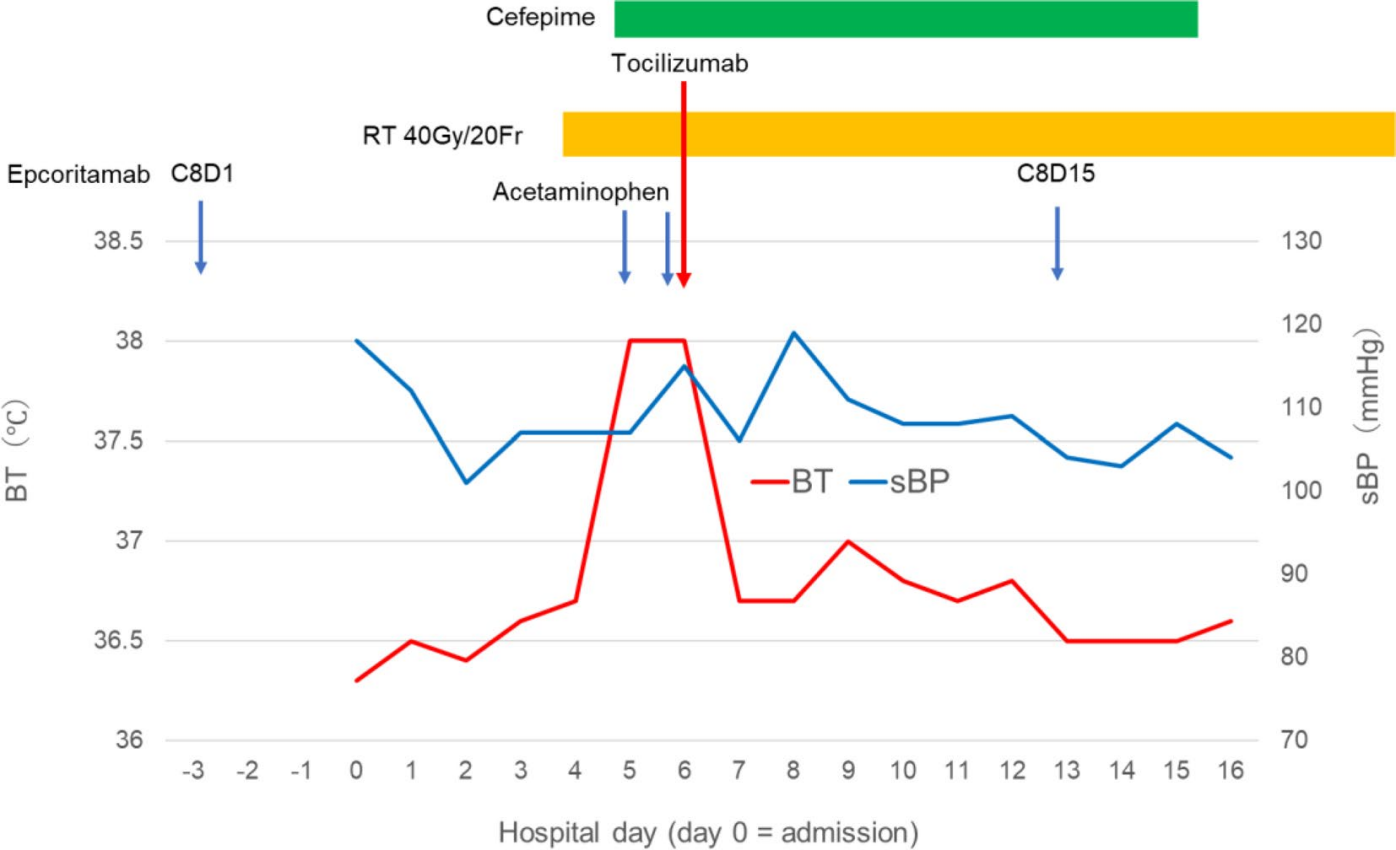
Clinical course during late-phase epcoritamab therapy and radiotherapy. Timeline of epcoritamab treatment, local radiotherapy (RT; 40 Gy in 20 fractions) for a residual abdominal nodal lesion, fever episodes, infectious workup, and clinical interventions. RT was initiated on epcoritamab cycle 8 day 8 (C8D8). The x-axis indicates hospital days (hospital day 0 corresponds to the day of admission). RT day numbering is based on the day of RT initiation (RT day 1 = first fraction). Within this timeline, the first and second post-initiation fever episodes occurred on RT day 2 and RT day 3, respectively (corresponding to the second and third RT fractions). Blood cultures were obtained, and empiric cefepime plus acetaminophen was initiated after the first fever episode, resulting in transient defervescence. After fever recurrence on RT day 3, tocilizumab (8 mg/kg) was administered for a CRS-compatible event, in accordance with standard CRS management, with defervescence within 1 h and no recurrent fever thereafter. Body temperature (BT) (daily maximum) and systolic blood pressure (sBP) (daily minimum) are plotted using values recorded in the electronic medical record and are shown to visualize the overall clinical course rather than exact event-time measurements. Abbreviations: RT, radiotherapy; BT, body temperature; sBP, systolic blood pressure; CRS, cytokine release syndrome

Epcoritamab was administered subcutaneously using a step-up dosing regimen: 0.16 mg on cycle 1 day 1 (C1D1), 0.8 mg on C1D8, and 48 mg on C1D15 and C1D22. Thereafter, 48 mg was administered weekly in cycles 2–3, every 2 weeks in cycles 4–9, and every 4 weeks from cycle 10 onward [6]. This step-up approach is intended to mitigate CRS risk [7, 8]. Concomitant prophylaxis included fluconazole, trimethoprim–sulfamethoxazole, and valacyclovir. The clinical course is summarized in Fig. 1, and selected laboratory data are shown in Table 1.

**Table 1:** **Footnotes (timing)**: Results were obtained at admission unless otherwise noted. The following were measured during hospitalization: galactomannan (GM) index and (1,3)-β-D-glucan (BDG). Creatinine clearance was estimated using the Cockcroft–Gault equation

Parameter	Value	Unit
Complete blood count		
WBC	6900	/µL
Neutrophils	65.3	%
Lymphocytes	18.7	%
Monocytes	9.3	%
Eosinophils	5.8	%
Basophils	0.9	%
Hb	13.9	g/dL
PLT	326 × 10^3^	/µL
Biochemistry		
Total bilirubin	0.3	mg/dL
AST	17	U/L
ALT	6	U/L
ALP	128	U/L
GGT	46	U/L
LDH	241	U/L
BUN	18	mg/dL
Serum creatinine	1.10	mg/dL
Creatinine clearance	49.4	mL/min
Calcium	9.1	mg/dL
CRP	2.86	mg/dL
Immunoglobulin		
IgG	495	mg/dL
Tumor markers		
soluble interleukin-2 receptor	1780	U/mL
Fungal markers		
Galactomannan (GM) index	0.1	—
(1,3)-β-D-glucan (BDG)	3	pg/mL

Abbreviations used in Table 1: ALP, alkaline phosphatase; ALT, alanine aminotransferase; AST, aspartate aminotransferase; BDG, (1,3)-β-D-glucan; BUN, blood urea nitrogen; CRP, C-reactive protein; GGT, gamma-glutamyl transferase; GM, galactomannan; Hb, hemoglobin; IgG, immunoglobulin G; LDH, lactate dehydrogenase; PLT, platelet count; WBC, white blood cell

On epcoritamab cycle 8 day 8 (C8D8), local RT (40 Gy in 20 fractions) was initiated for a residual abdominal nodal lesion. RT was started one week after the most recent epcoritamab dose in cycle 8 (C8D1), during the every-2-week dosing phase (cycles 4–9). Approximately 12 h after the second fraction (RT day 2), he developed fever (38.0 °C) and fatigue without hypotension, hypoxemia, or other localizing symptoms. Vital signs remained stable (no shock-level hypotension; SpO₂ ≥98% on room air; respiratory rate approximately 16/min). He had no respiratory symptoms, urinalysis was negative, and he had no indwelling central venous catheter, making line-related infection unlikely. Blood cultures were obtained, and empiric cefepime with acetaminophen was initiated, resulting in transient defervescence; however, fever recurred approximately 12 h after the third fraction (RT day 3). He remained hemodynamically stable without oxygen requirement and had no neutropenia. Blood cultures were negative, and serum (1,3)-β-D-glucan and galactomannan assays were negative, making invasive fungal infection unlikely. Given the reproducible timing after RT fractions and the clinical course, a CRS-compatible event (ASTCT grade 1) was considered; however, alternative causes of fever could not be completely excluded. Because the fever recurred in close temporal association with RT and there was concern that further progression might compromise the safe continuation of RT, tocilizumab (8 mg/kg) was administered on RT day 3 at the grade 1 stage based on ASTCT grading and clinical risk assessment, resulting in prompt resolution of fever within 1 h. He remained afebrile thereafter, and RT was continued to completion (40 Gy in 20 fractions) without recurrent fever.

### Discussion and conclusions

Clinical studies of epcoritamab monotherapy indicate that CRS is predominantly an early-event toxicity, clustering after the first full dose on cycle 1 day 15 and remaining largely low grade in LBCL. Extended follow-up suggests that de novo CRS is uncommon beyond the early cycles, with the latest reported onset at cycle 4 day 1 [2, 6]. One proposed explanation for the lower CRS frequency during later cycles is a priming effect with step-up and repeated dosing, which can attenuate cytokine release compared with initial exposure [4, 9].

Against this established temporal pattern, the reproducible fever that occurred approximately 12 h after RT fractions during cycle 8 suggests an immune-mediated process consistent with a CRS-compatible event. At the time of fever, vital signs were stable (no shock-level hypotension; SpO₂ ≥98% on room air; respiratory rate approximately 16/min), and there were no respiratory symptoms or indwelling central venous catheter; urinalysis was negative, making line-related infection less likely. An infectious workup was performed in parallel (including blood cultures), and no hemodynamic instability or hypoxemia was observed. Rapid defervescence within 1 h of tocilizumab is consistent with a cytokine-mediated mechanism, although viral testing and cytokine measurements were not performed and infection or other non-CRS causes of fever cannot be completely excluded.

Published reports of CRS or CRS-like inflammatory events temporally associated with RT remain very limited, particularly during CD3×CD20 bispecific antibody therapy. Most reported cases have arisen in other immunotherapy settings; Barker et al. described CRS after RT in a patient receiving anti–programmed cell death protein 1 immunotherapy, together with a review of previously reported cases [10]. Against this background, the present case is clinically informative because it occurred during late-phase epcoritamab therapy, recurred after consecutive RT fractions, and improved rapidly after IL-6 blockade, thereby illustrating a CRS-compatible febrile episode arising outside the usual early-cycle window of epcoritamab therapy.

Mechanistically, RT may have acted as an inflammatory trigger. Local tissue injury and immunogenic cell death can release inflammatory mediators and augment immune activation in some settings [11]. CRS-like inflammatory events temporally associated with RT have been described in other immunotherapy settings, with systemic symptoms recurring after irradiation and accompanied by transient increases in pro-inflammatory cytokines, including TNF-α and IL-6 [10]. Although the therapeutic context differs, these observations support the possibility that RT may transiently amplify cytokine-driven systemic symptoms in selected settings [10]. More broadly, RT can induce systemic immune effects beyond the irradiated field in some settings, particularly in the context of immunotherapy, as discussed in relation to the abscopal effect [12, 13]. In our patient, the irradiated abdominal lymph node may have contained residual target B cells. RT-induced tumor or tissue injury may have transiently augmented local inflammatory signaling during ongoing epcoritamab exposure, and a transient interaction with epcoritamab-mediated T-cell engagement may also have contributed to the febrile episode [6, 10, 11]. This interpretation is hypothesis-generating rather than confirmatory. Prior CAR T-cell therapy may have influenced the patient’s immune background; however, no CRS had occurred after CAR T-cell therapy in this case, and its contribution to the present event remains uncertain.

Clinically, in patients receiving BsAb therapy, fever developing during RT should prompt concurrent evaluation for infection while keeping CRS in the differential diagnosis [3–5]. Management should be guided by ASTCT criteria, with timely initiation of standard therapy, including IL-6 blockade when clinically warranted based on the overall risk assessment (e.g., recurrent fever with a reproducible temporal pattern and concern for deterioration that could compromise RT completion) [3–5]. A key limitation is that cytokine measurements were not obtained at symptom onset; therefore, causality cannot be established and the association remains clinical and temporal. However, in real-world practice, management decisions must often be made before cytokine data are available; thus, prompt parallel infectious workup and early CRS-directed management based on clinical grading remain essential when patients develop fever during BsAb therapy.

In conclusion, while CRS with epcoritamab is typically confined to early treatment, this case suggests that RT delivered during late-phase therapy may be associated with a CRS-compatible febrile episode. When RT is administered during BsAb therapy, clinicians should remain alert to CRS even in later cycles and manage suspected events promptly using established grading and management principles, including tocilizumab when clinically indicated.

## Data Availability

All data generated or analyzed during this study are included in this published article.

## Abbreviations

ASTCT: American Society for Transplantation and Cellular Therapy
BsAb: bispecific antibody
CAR: chimeric antigen receptor
CRS: cytokine release syndrome
DLBCL: diffuse large B-cell lymphoma
IL-6: interleukin-6
LBCL: large B-cell lymphoma
R/R: relapsed/refractory
RT: radiotherapy
TNF-α: tumor necrosis factor-α
